# Examination of pain relief effect of Goreisan for glossodynia

**DOI:** 10.1097/MD.0000000000021536

**Published:** 2020-08-14

**Authors:** Takao Ayuse, Ichiro Okayasu, Mizuki Tachi-Yoshida, Jun Sato, Hironori Saisu, Masahiko Shimada, Yoko Yamazaki, Hiroko Imura, Naoki Hosogaya, Sawako Nakashima

**Affiliations:** a Division of Clinical Physiology, Department of Translational Medical Sciences, Nagasaki University Graduate School of Biomedical Sciences; b Department of Dental Anesthesiology, Nagasaki University Hospital, Nagasaki; c Aichi Medical University, Nagoya; d Orofacial Pain Clinic, Tokyo Medical and Dental University Dental Hospital, Tokyo; e Nagasaki University Hospital, Clinical Research Center, Nagasaki, Japan.

**Keywords:** glossodynia, Goreisan, Kampo, pain-relieving effect

## Abstract

**Background:**

Pain in glossodynia may be severe; it may prevent patients from working, interfere with daily life activities, and necessitate a patient's visit to a medical institution for consultation and treatment. The pain may be described as persistent and burning (tingling, tingling) or stinging. Patients may complain of dry mouth (dryness), which is thought to cause inflammation of the tongue and gingival mucous membranes and increased pain. Medications are prescribed based on the symptoms of glossodynia, and the therapeutic effect is confirmed. However, each drug has side effects, for example, pain may reduce, but drowsiness and dizziness may occur; further, there is always a tendency of drowsiness.

On the other hand, Goreisan, a Chinese herbal medicine, has already been used by physicians to treat pain in the oral and maxillofacial regions resulting from rapid changes in air pressure. However, the lack of high-quality clinical research has been of concern, and a randomized clinical trial to investigate the efficacy and safety of Goreisan for treatment of pain in glossodynia is warranted.

**Methods/design:**

This multicenter, randomized, controlled study will involve patients treated for glossodynia-related pain. In the experimental group, Goreisan will be taken for 12 weeks in combination with conventional treatment. Participants in the control group will not take any Kampo medicine; only the standard treatment will be taken. Subsequently, the degree of pain will be assessed, and saliva tests of all the patients on their first visit will be performed. Goreisan will be taken at a dose of 7.5 g/d (minute 3) for 12 consecutive weeks. Twelve weeks later, the degree of pain of each patient will be assessed.

**Discussion:**

The purpose of this study is to investigate the efficacy of Goreisan for pain reduction in patients undergoing treatment for glossodynia-related pain. If pain in glossodynia patients can be reduced by the administration of Goreisan, its candidacy as an alternative treatment for pain in glossodynia can be further supported by more reliable research.

**Trial registration:**

The study was registered in the jRCTs071200017. URL https://jrct.niph.go.jp/latest-detail/jRCTs071200017.

## Abstract 2

Supplemental Digital Content is available in the text

## 1 Introduction

### 1.1 Pain of glossodynia

“Pain of glossodynia” is persistent and chronic pain that occurs in the mucosal surface of the mouth, and it is the same condition as “burning mouth syndrome.” Because of the name of the disease, it is often referred to as “tongue pain” including intra-oral burning. In the 3rd edition of the International Headache Classification, it is classified as one of the central facial pains and named as an intraoral burning syndrome. According to the 3rd edition of the International Headache Classification, glossodynia is defined as a tingling sensation in the mouth, a tingling sensation, or a tingling unpleasant sensation that recurs for more than 2 hours a day and daily for more than 3 months.

At present, there is no curative treatment for tongue pain because the cause has not been established. Therefore, symptomatic treatment has been used. To date, symptomatic treatments with reported benefits include cognitive-behavioral therapies and topical and oral therapies such as anticonvulsants. Many patients complain of pain during the treatment of glossodynia over a long period; instead of the elimination of pain by the therapies, they end up managing and overcoming the pain that is interfering with their daily life activities.

### 1.2 Characteristics of the pain of glossodynia

The pain of glossodynia is all the pain that a patient feels, and it is difficult for others to understand because it is not abnormal even when viewed by others. The degree of pain in glossodynia is sometimes severe and may interfere with work and daily life activities, and necessitate a visit to a medical institution. The nature of the perceived pain may be described as persistent and burning (tingling, tingling) or stinging (tingling, throbbing). It usually lasts from wake-up time to bedtime, but the intensity of the pain has a wave, and it usually does not prevent sleep. Pain is closely linked to psychosocial stress, and anxiety and discomfort at work and home can exacerbate it.

In addition, patients with tongue pain often complain of dry mouth (dryness), which is thought to cause inflammation of the tongue and gingival mucous membranes, worsening the pain. The relationship between taking drugs that suppress oral respiration and the secretion of saliva and stress has also been highlighted, and taste disorders are known as accompanying symptoms. Some case reports revealed that tongue pain was reduced by treating dry mouth.^[1–3]^ Sugiyama et al^[2]^ reported that the symptoms of dry mouth and tongue pain in a 75-year-old male resolved approximately 2 weeks after receiving Goreisan as a treatment for hydrosis when he presented with a chief complaint of dry mouth. Therefore, treating dry mouth may reduce tongue pain.

Various medications such as pregabalin, antiepileptic drugs, duloxetine, amitriptin, antidepressants, N-Methyl-D-aspartate receptor antagonists, and tramadol are prescribed depending on the symptoms of glossodynia. Although pain may reduce, each drug might have side effects, such as drowsiness and dizziness.^[4]^ (II. Pharmacotherapy, CQ8-CQ21, Guidelines for the treatment of chronic pain, 2018)

On the other hand, Goreisan, a Chinese herbal medicine, has already been used by physicians to treat glossodynia and other forms of pain in the oral and maxillofacial regions following weather conditions including change in the barometric pressure. There have been several reports that the administration of Goreisan was effective for pain in glossodynia patients^[5–8]^ caused by acute changes in barometric pressure. Furthermore, large-scale epidemiological studies have shown that pain in rheumatoid arthritis is exacerbated by a weather storm (particularly due to a decrease in air pressure 3 days before the pain test). In the oral and maxillofacial regions, many tissues, including the tongue, are rich in peripheral capillaries and lymphatic vessels and are susceptible to changes in body fluid balance.

As described above, Goreisan may promote the metabolism of tissues such as local mucous membranes, improve stasis, and improve the flow of lymph, which may improve chronic pain. The administration of Goreisan is considered to improve symptoms of the tongue; the tongue is rich in vascular tissue and its symptoms only have few accompanying symptoms of the central nervous system, such as drowsiness and dizziness, and they are associated with a low risk of liver and renal dysfunction. However, as indicated in the latest guidelines for the treatment of chronic pain, the level of evidence for the clinical question, “Is Chinese medicine effective for treating chronic pain?,” remains as 2C to 2D. It is said that no high-quality clinical research has been conducted. In addition, although Kampo medicines are not the same as Western medicines, there is a risk of the side effects of the constituent crude ingredients and cautionary points on interactions with concomitant drugs, and a thorough investigation and a well-designed clinical study are required.

In this study, we will also explore changes in weather (barometric pressure and temperature) that may affect pain perception. Some patients suffering from chronic and persistent pain sometimes complain as follows: “Before it rains, pain in the tongue becomes worse.” The reason for the worsening of pain when the weather changes is not completely understood; it is thought to be caused by a malfunction of sensors that detect atmospheric pressure in the body. Traditionally, oriental medicine has a meteorological medicine sub-field, and when a condition worsens due to a specific weather condition, therapy corresponding to the condition is applied; this has been practiced since ancient times. It is also known that a decrease in barometric pressure exacerbates pain in the trigeminal nerve distributions, including headache in the oral and maxillofacial region and pain in the oral cavity.

It has been suggested that the reason for the worsening of chronic pain caused by meteorological diseases is the activation of the sympathetic nervous system by a signal from a sensor, such as the inner ear, that senses atmospheric pressure. This stimulates stress and causes various diseases. There is also a theory that the pressure of the human body is reduced following the decrease in atmospheric pressure, and the blood vessels easily dilate accompanied by the stagnation of lymph. However, there are only a few basic studies on meteorological diseases and the details are not clear. Sato and colleagues report that changes in the weather associated with a decrease in barometric pressure exacerbate the pain associated with meteorological illness, and they have shown in animal experiments that different neurons in the vestibular nucleus respond to changes in air pressure.^[9]^ A similar mechanism has been observed in humans, and this suggests a link between human meteorological diseases and pain caused by changes in atmospheric pressure.^[10,11]^ If Goreisan can reduce pain in glossodynia patients, it will open up new drug opportunities. There are strong claims that Goreisan is effective for tongue pain, and it cannot be denied that it is one of the effective treatments. However, until now, there is little reliable evidence on the efficacy of Goreisan in reducing pain. In this study, we investigate the tongue pain-reducing effect of Goreisan. In this study, we will also explore the association between the changes in weather (barometric pressure and temperature) and pain. The purpose of this study is to investigate whether Goreisan can reduce pain in patients undergoing treatment for tongue pain. If Goreisan can reduce pain, its candidacy as an alternative treatment for pain in glossodynia can be further supported by more reliable research.

## 2 Methods/design

### 2.1 Study design

This multicenter, randomized, group-controlled study will involve patients treated for glossodynia. Patients with consent will be randomized and assigned to 1 of 2 groups as shown below (ratio 1:1). In the experimental group, Goreisan will be taken for 12 weeks in combination with conventional treatment, Patients in the control group will only receive the standard treatment (Western medicine administration). The study will be conducted mainly at the Nagasaki University Hospital in Japan. It is registered on the Japan Registry of Clinical Trials. It will also be conducted following the principles of the Declaration of Helsinki and the established best clinical practices of Japan.

### 2.2 Participant recruitment

Participants have been recruited from the Nagasaki University Hospital, where the subsequent study will also take place. The treating clinical research coordinator explained the study to all participants, and each participant signed an informed consent form. This is intended for patients undergoing medical treatment with the chief complaint of glossodynia. The medical institutions that will conduct research will target patients undergoing medical treatment at Nagasaki University Hospital (Oral Pain Liaison Outpatient center), Aichi Medical University Hospital, and Tokyo Medical and Dental University. The case registration and allocation organizations are Nagasaki University Hospital, Tokyo Medical and Dental University, and Aichi Medical University; each will perform case registration and allocation using REDCap. REDCap was used to randomly allocate participants to the “Goreisan-administration” and “non-Goreisan-administration” groups at a ratio of 1:1 (stratified block method).

#### 2.2.1 Sample size

In this study, ninety patients with the chief complaint of glossodynia undergoing medical treatment are targeted. A preliminary study (Okayasu I. et al, Advances in Traditional Medicine. 2020, In press) found that after 2 weeks of Goreisan administration, the degree of pain on visual analog scale (VAS) was reduced by more than 20%. Improvement was observed in 8 of 14 cases (about 57%). On the rate of pain improvement, the effective rate of the standard treatment group is 10% and the expected effective rate of this drug is 40%, with α of 0.05 and power of 0.9. The number of cases should be approximately 82 (41 in each group) because statistical analysis is possible in approximately 90 cases, in considering a dropout of approximately 10%. Therefore, the sample size is set for approximately 90 cases in total with 45 cases in each group. Each of the 3 medical institutions will be assigned 30 cases (15 cases per group).

### 2.3 Inclusion criteria

The following were the inclusion criteria:

(1) Patients undergoing treatment for chronic pain in the oral and maxillofacial region.
(2) Age: At the time of obtaining consent, an adult patient who is 20 years or older.
(3) Gender: Any.
(4) Hospitalization/Outpatient: Outpatient only.
(5) Patients who have been given sufficient explanations, have sufficient understanding to participate in this study, and have provided informed consent.

### 2.4 Exclusion criteria

The exclusion criteria were as follows:

(1) Patients taking Kampo medicines including Goreisan.
(2) Severe hypertension with a diastolic blood pressure of 120 mm Hg or more.
(3) Patients with severe liver and renal dysfunction.
(4) Patients with drug hypersensitivity.
(5) Pregnant women and patients who may be pregnant or lactating.
(6) Patients who participated in another clinical study within 4 months.
(7) Other patients judged by the investigator to be ineligible as study subjects.

### 2.5 Study protocol

Patients who have provided consent should be visited in advance, and the degree of pain and saliva at that time will be assessed. After that, Goreisan will be taken at 7.5 g/d (minute 3) for 12 consecutive weeks. Four weeks later, the patients will return to the hospital for a second assessment of pain and saliva. Eight weeks after this, the patients will be reexamined, and the third assessment of pain and saliva will be performed. Twelve weeks after the first test, a fourth test will be performed.

During each outpatient consultation, the weather conditions, such as temperature, humidity, and barometric pressure, should be recorded and displayed on the outpatient meteorometer on the chart. Patients should also observe, and possibly record, the degree of pain at home, in addition to assessments during their visit to the outpatient clinic at a medical institution (Table 1).

**Table 1:**
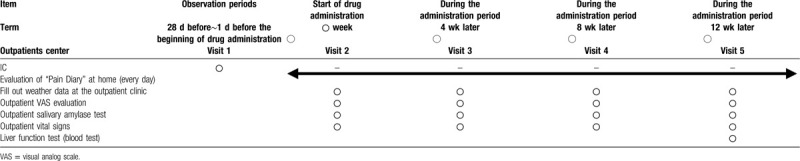
Research schedle and items.

### 2.6 Adverse events

In this study, adverse events related to the study drug (possible liver dysfunction due to long-term use of Goreisan) will be considered. During the study period, blood will be drawn and liver function tests will be performed. If there are any abnormalities, the study will be discontinued immediately and appropriate treatment will be administered.

### 2.7 Outcome

The primary endpoint is the 20% or more improvement on the VAS relative to the control value at the outpatient visit 12 weeks after administration based on the self-filled VAS of patients.

(The main evaluation point is the 20% or more improvement in the VAS control value at the outpatient visit 12 weeks after administration.) (All itmes to be collected as listed in SDC).

#### 2.7.1 Rationale for an improvement rate of 20% or more

The degree of pain will be evaluated using VAS. Regarding the determination of VAS based on subjective symptoms, those with a decrease of 20% or more are “improved,” and the other cases are “not improved.”

The grounds for improving the VAS entered by the patient by 20% or more are as follows. This criterion was based on previous drug information for celecoxib (KEGG Drug Databese), using the 20% improvement criterion used by the American College of Rheumatology. For this criterion, patients with a 20% or greater improvement in pain perception (VAS) were considered valid cases when assessing the efficacy of celecoxib. In the clinical trial that evaluated the clinical outcomes of celecoxib for rheumatoid arthritis pain, assessments were performed using the American College of Rheumatology improvement rates (various methods), and the results provided drug information for treating patients with a VAS improvement of 20% or more. This standard was adopted for pain improvement following the administration of Goreisan.

#### 2.7.2 Secondary outcome

The secondary endpoint is improvement of each item at the outpatient visit at 4 and 8 weeks after Goreisan administration.

(1) VAS evaluation
(2) Salivary amylase activity (10–200 kIU/L)
(3) Tongue examination (whether the veins on the back of the tongue are angry)
(4) Weather information (barometric pressure)

### 2.8 Efficacy

Efficacy will be evaluated using a 20% or more improvement on the VAS relative to the control value at the outpatient visit 12 weeks after administration based on the self-filled VAS of patients.

(The main evaluation point is the improvement rate of 20% or more of the VAS control value at the outpatient visit 12 weeks after administration.)

### 2.9 Safety

The safety evaluation indices of this clinical trial are as follows: adverse events are any undesired or unintended signs (including abnormal laboratory values, abnormal vital signs), symptoms, or illnesses that occur between the beginning of Goreisan use and the end of the last observational study. It does not matter whether the study demonstrates a causal relationship. Symptoms and diseases occurring before the use of medical devices are treated as complications and not adverse events. However, if the complications worsen after the beginning of the medical device use, they will be treated as adverse events, and the day on which the deterioration is confirmed will be the date of occurrence of the adverse events. After enrolment, if the patient discontinues taking the drug for 12 weeks during the administration of Goreisan, the study should be discontinued.

### 2.10 Data collection and management

The assignment and input tables to be used in this study were created with the REDCap. The study will be conducted after allocating registered patients. The data on all items in the medical records collected in the study will be assigned to the researcher who will be assigned the ID entered by a physician, co-doctor, and co-worker. The Principal Investigator or Co-Researcher will approve the input observation/inspection/evaluation data of each research subject immediately after confirming the content. For the data entered in the case report, the Principal Investigator and the Clinical Research Center Data Management staff will perform visual and logical checks. If there are any problems or doubts in the data after each check, the principal investigator or the research coordinator should be contacted. A data lock will be performed on the case when the issue has been resolved and all modifications have been completed. If there is an error that needs to be corrected after the case is locked, the data management staff is responsible for overseeing this process. Goreisan Supplement Items.

### 2.11 Statistical analysis

The difference between the improvement rate between the Goreisan administration group and the non-administration standard treatment group will be analyzed by Fisher exact test. Test the null hypothesis: there is no difference between the rates of improvement of the Goreisan administration group and the standard treatment group.

In addition, the degree of pain will be evaluated using VAS. The verdict for VAS based on subjective symptoms, which is reduced by 20% or more, is “improved,” and the others are “non-improved.” The grounds for improving a VAS that has been reduced by more than 20% from the target value entered by the patient are as follows.

## 3 Discussion

Goreisan, a Chinese herbal medicine, has already been used by Kampo and physicians to treat pain in the oral and maxillofacial regions due to rapid changes in air pressure. To date, no high-quality randomized clinical trial has investigated the efficacy of Goreisan in reducing pain in glossodynia patients. The guideline strongly recommended that further investigations of efficacy and safety of Kampo medicine in the treatment for chronic pain possibly related to the change in the weather condition. In addition, although Kampo medicines are different from Western medicines, there is a risk of the side effects of constituent crude ingredients and interactions with concomitant drugs. Hence, a thorough investigation and a well-designed clinical study are required. This clinical trial will be the first study to ascertain the efficacy and safety of Goreisan for the treatment of pain in glossodynia patients.

## Acknowledgments

The authors would like to thank our colleagues and staff at the Dental Anesthesiology Department of Nagasaki University Hospital for their support.

## Author contributions

TA, JS, NH, SN are responsible for conceiving and designing the trial, planning data analysis, drafting the manuscript, and approving the final manuscript. JS, IO, MT, and MS are responsible for preparing all the evaluation tools including the VAS daily memo. JS, MS, IO, and MT will participate in data collection and are in charge of the recruitment and treatment of patients. SS and SM are responsible for planning data analysis and analyzing the data resulting from the trial. All authors will have access to the interim results as well as the capacity to discuss, revise, and approve the final manuscript.

## Supplementary Material

Supplemental Digital Content
