# Supplementary material for: Examination of pain relief effect of Goreisan for glossodynia
Source: Medicine (Baltimore). 2020 Aug 14;99(33):e21536. doi: 10.1097/MD.0000000000021536 (PMC7437797; doi:10.1097/MD.0000000000021536)
Supplement: Supplemental Digital Content [file medi-99-e21536-s001.docx]

Items to be collected by the doctor when the patient visits:

(Enter data in REDCap and manage data)

(Pre-observation period)

1. Patient background: gender, age, height, weight, medical history, complications, and concomitant medication
2. Background of glossodynia: Severity assessment by VAS, site, past treatment contents, current combination therapy

(Start date of administration, 4 weeks, 8 weeks, 12 weeks after administration)

1. VAS evaluation (at each visit, the patient fills in the VAS at the time of meeting with the medical staff)
2. Meteorological data (At each visit, the person in charge measures the atmospheric pressure with an outpatient device and enters it)
3. Collect saliva of the patient, perform saliva amylase test (10-200kIU / L), and enter the data
4. Perform a tongue examination (fill in the veins on the back of the tongue whether it is angry)
5. Enter the presence or absence of stagnant water
6. Vital signs (blood pressure, pulse rate, respiratory rate); enter any adverse events (during administration)
7. Liver function test (AST, ALT, γ-GTP)

Items to be collected by patients using their individual "Pain Diaries" at home

(record in each patient's Pain Diary, save it as an original book, and enter the data in a common Excel sheet after the study to manage the data).

1. Date and time (Monday / Sunday)
2. Weather information (clear, cloudy, rain, etc.)
3. The drug that patients started taking (the drug that patinets stopped taking)
4. Patient's self-record (what they noticed, the behavior of the day, etc.)
5. VAS evaluation of pain (patient fills in the VAS for the day by himself/herself: both strongest pain and weakest pain in the day are written in VAS)
6. Exercise (exercises or not)
7. Sleep state (good sleep, poor sleep, bad sleep)
